# The Clinical Value and Interpretation of Anti-Müllerian Hormone in Women With Cancer

**DOI:** 10.3389/fendo.2020.574263

**Published:** 2020-10-07

**Authors:** Richard A. Anderson, H. Irene Su

**Affiliations:** ^1^ MRC Centre for Reproductive Health, Queen’s Medical Research Institute, University of Edinburgh, Edinburgh, United Kingdom; ^2^ Department of Obstetrics, Gynecology and Reproductive Sciences, University of California, San Diego, La Jolla, CA, United States

**Keywords:** ovarian reserve, cancer, anti-Müllerian hormone, fertility preservation, premature ovarian insufficiency

## Abstract

Cancer treatments can be damaging to the ovary, with implications for future fertility and reproductive lifespan. There is therefore a need for a biomarker than can usefully provide an assessment of the ovary and its potential for long-term function after cancer treatment, and ideally also be of value pre-treatment, for the prediction of post-treatment function. In this review we assess the value of anti-Müllerian hormone (AMH) in this context. Measurement of AMH at the time of cancer diagnosis has been shown to be predictive of whether or not there will remain some ovarian function post-treatment in women with breast cancer, in conjunction with age. AMH may however be reduced at the time of diagnosis in some conditions, including lymphoma, but probably not in women with breast cancer unless they are carriers of *BRCA1* mutations. Following chemotherapy, AMH is often much reduced compared to pretreatment levels, with recovery dependent on the chemotherapy regimen administered, the woman’s age, and her pretreatment AMH. Recent data show there may be a long duration of relative stability of AMH levels over 10 to 15 years prior to decline rather than a rapid decline for many young women after cancer. Post-treatment AMH may have utility in determining that ovarian function will not recover, contributing to assessment of the need for ovarian suppression in women with hormone-sensitive breast cancer. AMH measurement provides an index of treatment gonadotoxicity, allowing comparison of different treatment regimens, although extrapolation to effects on fertility requires caution, and there are very limited data regarding the use of AMH to estimate time to menopause in the post-cancer setting.

## Introduction

Adolescent and young adult cancer survivors can experience many late effects related to their cancer treatment. With substantial improvements in survival following many common cancers, perhaps most importantly in the present context for women with breast cancer (1), and recognition of the importance of quality of life following treatment, identifying, and treating reproductive and sexual health late effects has become increasingly important for these young women. While not all cancer treatments adversely impact the ovary, the overall likelihood of a woman having a pregnancy following cancer treatment is reduced by nearly 40% across all diagnoses (2). Cancer survivors express considerable unmet informational needs on individualized risks of adverse sexual and reproductive health outcomes and clinical management options, contributing to lower quality of life and distress (3, 4).

Ovarian function, specifically the development of ovarian follicles and associated reproductive hormones, contributes directly to reproductive and sexual health in this population. Measures of ovarian function for diagnostic and predictive indications may help clinicians and patients understand their current as well as predicted future ovarian function. While conventional indicators of ovarian function, namely cyclical menstruation, gonadotropins, estradiol, and progesterone remain essential for assessing current ovarian function (growth of larger follicles and ovulation), the last 15 years have seen a substantial interest in the potential value of measuring anti-Müllerian hormone (AMH). The objective of this review is to discuss current understanding of the clinical value of measuring AMH in young cancer survivors at cancer diagnosis and post-treatment. Specific questions we address include whether AMH levels at cancer diagnosis and/or post-treatment predict response to ovarian stimulation in the short-term, and fertility or time to menopause in the long-term. We also discuss how AMH levels reflect current ovarian function and help to estimate the gonadotoxicity of cancer treatments.

Other reviews in this collection will address many of the details surrounding the origins of AMH and what is known about its role and value in normal ovarian physiology. For the present context, the key points are that AMH is not produced by primordial follicles, but it is produced by the granulosa cells of growing preantral and small antral follicles. There is a relationship between the number of primordial follicles and the number of growing follicles in the adult human ovary (5), and the limited data available show that AMH levels correlate with the number of primordial follicles (6). Thus, AMH is an indirect marker of the true ovarian reserve, i.e., the number of non-renewable, non-growing primordial follicles. Importantly, AMH production by granulosa cells falls dramatically when follicles reach a diameter of approximately 10 mm, and it is estimated that follicles of 5 to 8 mm diameter contributes the majority of circulating AMH (7). These follicles will have been in the growth phase for a significant period of time, probably many weeks, and are approaching a key timepoint when they may or may not be selected for dominance and ovulation, with the great majority, as at all stages of follicle growth, destined for atresia. Thus, AMH may be regarded as a reflection of what can be termed the functional ovarian reserve, which is those follicles which are starting to produce estrogen and in contributing to basal, early follicular, estrogen production, and underpinning the potential for ovulation. This is, of course, the basis for the value of AMH-based treatment strategies in assisted reproduction, as an index of follicular response to ovarian stimulation.

A further important attribute of AMH is that is it detectable in the circulation in childhood as well as in adults, though with complex age-dependent changes (8, 9). Specifically, after a temporary neonatal peak, AMH levels are initially low in childhood, rising to a plateau after puberty until the mid 20s, then progressively decline thereafter to undetectable levels associated with the menopause. It is important to recognize that these physiological changes in AMH levels may result in a peri-pubertal decline and then rise in adolescent girls, which complicates interpretation during puberty and the years thereafter. While the relationship between AMH and the ovarian reserve thus changes between childhood, adolescence and early adulthood, and the main reproductive years (10), the measurement of AMH allows some assessment of ovarian function in prepubertal girls as well as young adults.

## Measuring AMH Before Cancer Treatment

The measurement of AMH at the time of diagnosis in women with cancer has two important clinical uses. Typically measured in clinics, most immediately, it is of value in assessing the functional ovarian reserve in women considering ovarian stimulation for fertility for egg or embryo vitrification for fertility preservation, and therefore the question is whether it has the same predictive value as in the normal situation in women having assisted reproduction. Second, in conjunction with age and cancer treatment, it may be of value in predicting long-term ovarian function after cancer treatment is completed.

### AMH and Ovarian Stimulation for Fertility Preservation Before Cancer Treatment

Data showing that AMH levels are reduced in women with lymphoma at the time of diagnosis compared to age match controls (11, 12). This may reflect the systemic inflammatory nature of lymphoma compared to other cancers, as AMH levels do not appear to be reduced in women with breast cancer (13, 14). Higher AMH predicts higher oocyte yield in ovarian stimulation of cancer patients (15). Overall, the results of ovarian stimulation with regard to number of oocytes retrieved and proportion fertilized are similar in women with cancer to women without cancer (16). There is, however, evidence of reduced oocyte quality compared to women cryopreserving oocytes for elective purposes (17), which is not reflected by AMH.

### AMH in Women With BRCA Mutations

A special situation in the context of breast cancer is the potential impact of mutations in the *BRCA1* and *BRCA2* genes on ovarian function. These genes encode proteins involved in the DNA damage repair pathway, which is of key importance in the oocyte (18), and there is good evidence from animal models that *BRCA1* in particular is necessary for normal fertility and ovarian lifespan (19). That study also suggested that women with *BRCA1* mutations also had lower AMH levels, and a reduced response to ovarian stimulation, and *BRCA1/2* carriage has been linked with an earlier age at natural menopause (20). It appears that women with *BRCA1* mutations, but probably not those with *BRCA2* mutations, do have a lower AMH level overall; it was found to be 25% lower in a study including 172 *BRCA1* mutation carriers (21), who were also more likely to have AMH levels in the lowest quartile (odds ratio 1.84, 95% CI: 1.11–303). Comparably, a reduced response to ovarian stimulation has also been reported in a cohort of *BRCA1* mutation carriers, with no effect in *BRCA2* mutation carriers (22). Others have found that AMH levels are not reduced in *BRCA* mutation carriers (23, 24), but those studies did not separately analyze *BRCA1* and *BRCA2* carriers.

### AMH at Cancer Diagnosis and Prediction of Long-Term Ovarian Function

**Figure 1:**
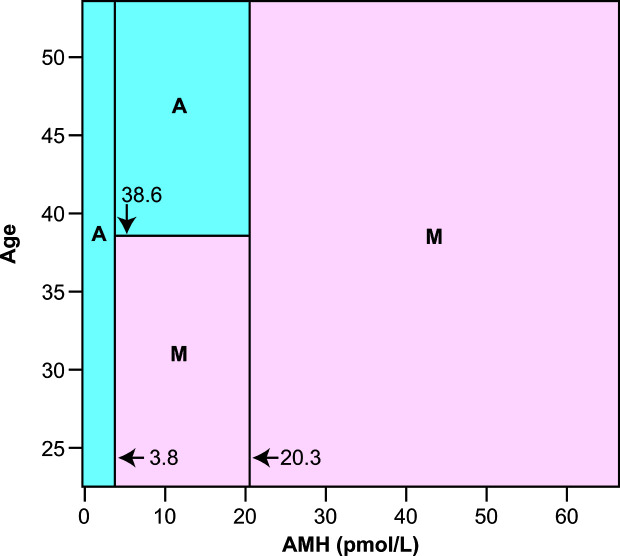
Classification mosaic chart for ongoing menses (M) or chemotherapy-related amenorrhea (A) at 2 to 5 years after diagnosis using serum AMH and chronological age at cancer diagnosis as predictor variables. The primary cutoff values are both for AMH, with below 3.8 pmol/L predicting amenorrhea and above 20.3 pmol/L predicting ongoing menses. Between these AMH levels there is an age threshold at 38.6 years, above which amenorrhea is predicted and below which ongoing menses are predicted. The classification schema has sensitivity 98·.2% (1 of 55 subjects known to have developed amenorrhea misclassified as having ongoing menses) and specificity 80.0% (4 of 20 subjects with known ongoing menses misclassified as amenorrhoeic). After 10-fold cross-validation this schema represents the optimal compromise between good fit to the data used to construct it, and low estimated error when used as a predictive model. Reprinted from (27) with permission.

The value of AMH measurement at the time of diagnosis in predicting long term ovarian function has been clearly demonstrated in women with breast cancer, with limited data for women with other diagnoses. Studies require a long-term prospective cohort recruited at the time of diagnosis, and are therefore relatively few in number. In women with breast cancer, pretreatment AMH predicts long term ovarian function measured as ongoing menses or not. In the first such analysis AMH was shown to have a better predictive value than age (25, 26), although the latter is also, of course, an important predictive factor. In a second similar prospective cohort, it was shown that pretreatment AMH below the median value for the group, 0.46 ng/ml, accurately predicted amenorrhea in all women at 2 years following diagnosis (27). Combining these two cohorts allowed production of a mosaic chart showing the interaction between age and AMH and pretreatment AMH in predicting whether or not the woman was likely to have long term amenorrhea at—2 to 5 years after diagnosis (**Figure 1**). Subsequently larger cohorts followed prospectively from diagnosis within breast cancer treatment trials (28) and specifically recruited have produced confirmatory data (29–31). In survivors of cancers other than breast cancer, it has been shown that pretreatment AMH impacts on the rate of recovery of AMH after chemotherapy, with higher pretreatment AMH associated with more rapid recovery (32). Importantly, the menstrual and AMH outcomes of these studies are limited in part by heterogeneity of definition on duration and timing after treatment, but indicate ovarian function and estrogen production, important to sexual and bone health. To date, however, no studies have investigated how pre-treatment AMH levels are related to post-treatment fertility or time to menopause in women with preserved ovarian function after cancer treatment.

## Measuring AMH After Cancer Treatment

### AMH Is a Measure of Current Ovarian Function in Post-Treatment Cancer Survivors

It is recognized that recovery of ovarian function after chemotherapy (as reflected in resumption of menses) varies by age and by diagnosis/treatment (33, 34). Younger women show a more rapid recovery, as do those treated for lymphoma compared to breast cancer, and recovery can take 2 years, or occasionally longer. AMH levels show this recovery. When modeling AMH in cancer survivors, levels are initially low immediately post-treatment then rise to peak between 2 and 3 years later (35). Prospective studies in women with breast cancer (median age 41), as discussed above, show both a marked fall versus pretreatment levels and minimal recovery of AMH levels over several years thereafter (25), while a comparable study in younger women with lymphoma (mean age 24) showed a clear divergence of the pattern of AMH levels by different chemotherapy regimens, with robust recovery of AMH levels in women treated with ABVD versus very limited recovery in women treated with high doses of alkylating agents (36).

With AMH levels drawn post-treatment as a reflection of current ovarian function in those patients, age, AMH at the end of treatment, and BMI are factors associated with the rate and extent of recovery in AMH following treatment. In addition to younger age, having a higher AMH at the end of treatment is associated with greater and faster recovery (32), and higher BMI may also be related to shorter time to recovery (31).

Following recovery after cancer treatment, AMH will again decline as it does in all women. Although this is a challenging aspect of the subject to study, whether the rate of decline is affected by prior chemotherapy has been investigated. In an analysis of the rate of decline of AMH in 170 cancer survivors aged 15 to 39 years over a 2-year period, the rate of decline in AMH was similar to that in similarly aged controls, albeit at lower levels overall, and in both younger groups was much slower than in older women, aged 40 to 50 years (37). A second study reported the slope of change in AMH over approximately 3 years in long-term childhood cancer survivors (median of 16 years since cancer treatment) was similar to women without cancer (38). Taken together, although AMH levels will on average be lower in a cancer survivor than a woman without cancer, there may be a long duration of relative stability with a plateau over 10 to 15 years prior to decline rather than a rapid decline for many young women who are cancer survivors (35). This is very reassuring for such patients, although additional detail is needed to confirm that this applies across the range of AMH levels.

### The Relationship Between Cancer Treatment and Ovarian Function is Reflected by AMH, and Is Modified by Age

**Figure 2:**
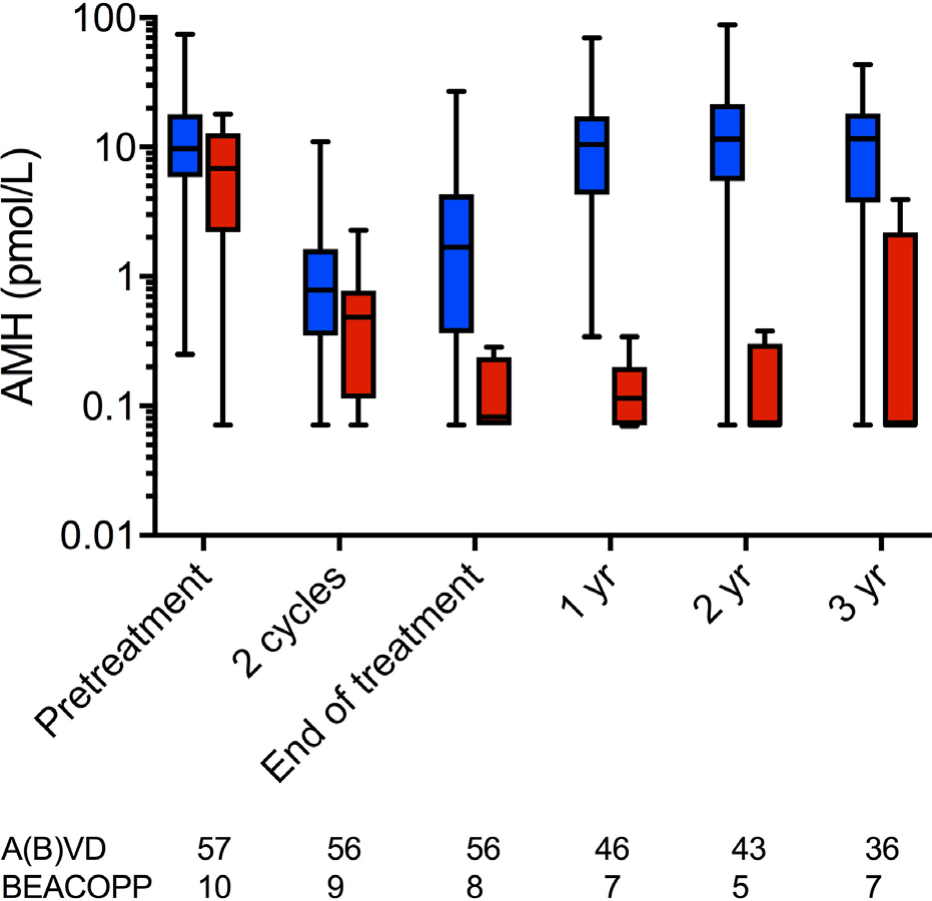
AMH concentrations at pre-chemotherapy, after two cycles of ABVD, at end of treatment and at 1, 2, and 3 years after chemotherapy. Blue: women treated with ABVD throughout; red, women treated with BEACOPP after two cycles of ABVD. Boxes are median and IQR, whiskers indicate range. AMH is plotted on a log10 scale to show the low concentrations during and after BEACOPP more clearly. Numbers of subjects at each time point indicated below. Reprinted from (39) with permission.

In a recent analysis of recovery of ovarian function following treatment for Hodgkin lymphoma, it was confirmed that women treated with ABVD overall showed a complete recovery of AMH levels, in contrast to those treated with BEACOPP (**Figure 2**) (39). However, within the ABVD treated group recovery of AMH levels was markedly reduced to approximately 35% of pretreatment values in women aged over 35, whereas it was complete in younger women. This was not related to pretreatment AMH levels, thus young women with a low AMH at diagnosis showed a good recovery whereas older women with a higher AMH did not, thus the effects of age may be indicative of other aspects of ovarian aging, perhaps affecting the stroma or vasculature, and their damage by chemotherapy (40). The effect of age on recovery of ovarian function was also demonstrated in FSH levels, which were slower to return to normal in women aged over 35 than in younger women (39). This differential relationship between chemotherapy and post-treatment AMH levels by age in women with Hodgkin lymphoma is in contrast to data in women with breast cancer, where no effect modification has been observed. Potential explanations include the overall older age of the breast cancer population, with the majority in their late 30s and early 40s in most studies, or it may reflect the gonadotoxicity of chemotherapy with overall much less recovery of AMH in women treated with breast cancer chemotherapy regimens.

Indeed, age modified the relationship between gonadotoxicity and post-treatment AMH trajectories in a large cancer survivor cohort (35). The trajectory for high gonadotoxicity (high dose alkylators, pelvic radiation, transplant) had a noticeably steeper decline after its initial rise in the first 2 to 3 years since cancer treatment, compared to the trajectories of the moderate and low gonadotoxicity groups which showed a prolonged plateau. In the same cohort, survivors who were older than age 30 at diagnosis exhibited consistently lower AMH trajectories compared to those younger than 25 years and those between ages 25 and 30 years. A test of interaction between age at treatment and gonadotoxicity was statistically significant, and data suggested that the protective effects of younger age on ovarian reserve when exposed to high gonadotoxicity treatments becomes diminished in the latter 20s.

Importantly, in the present context, recovery of AMH after chemotherapy does not reflect an increase in the ovarian reserve, but a recovery in the population of growing (AMH-producing) follicles in the functional ovarian reserve. Similarly AMH levels are reduced in healthy women taking hormonal contraception (41), and are also reduced in women with some cancer diagnoses, before treatment as discussed above.

### Acute and Long-Term AMH Levels as Measures of Cancer Treatment Gonadotoxicity

Many cross-sectional studies clearly show that treatment type is related to AMH levels, both acutely and over the long-term. The first such study to show this was in young women who had been treated for childhood cancer, but who still had regular menstrual cycles thus overtly had normal ovarian function (42). These women were shown to have reduced AMH levels compared to age match controls and this key finding of the added value of AMH in assessment of post-treatment ovarian reserve has subsequently been replicated in a large number of studies across a range of diagnoses. Subsequent studies showed that AMH was markedly low following treatment for breast cancer (43, 44), following alkylating agent therapy and treatments associated with bone marrow transplantation (45), in women treated for Hodgkin lymphoma in childhood where a relationship with dose of alkylating agent was identified (46), and across a range of diagnoses in childhood and young adult cancer survivors (47–50), confirming relationships with dose of alkylating agent and pelvic radiotherapy. The limitation of cross-sectional studies is that they shed little light on the pattern of change of ovarian function post treatment.

More limited prospective data are available. In a pediatric cohort of 22 girls with a range of diagnoses and aged 0.3 to 14 years, AMH fell progressively with each course of chemotherapy becoming undetectable in approximately half the group (51). The key finding was that initial fall of AMH levels and recovery varied by treatment regimen, with those treated with regimens assessed as having low/moderate gonadotoxicity showing recovery to pretreatment AMH levels, whereas those treated with high risk regimens (containing high doses of alkylating agents, or with pelvic radiotherapy) showed lower levels at the end of treatment and minimal or no recovery. Inhibin B and FSH were of no value in discriminating these treatment effects. This may allow improved assessment of girls at pre- and peri-pubertal ages, and support timely treatment for induction of puberty where there is clear and early evidence of absent ovarian function.

**Figure 3:**
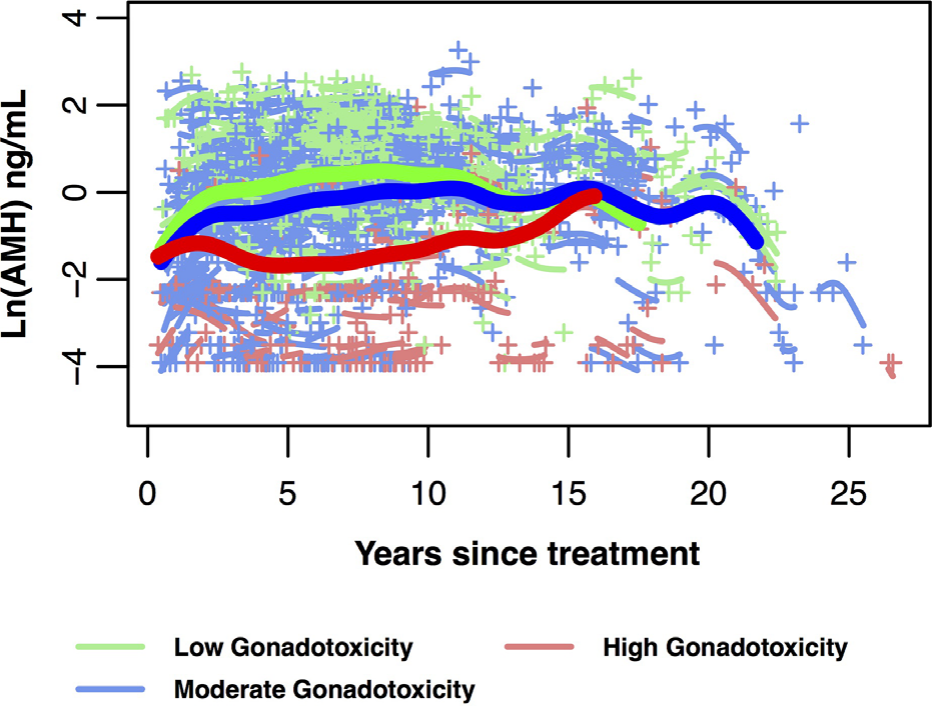
AMH trajectories in 718 post-treatment AYA cancer survivors ages 25 to 40 years at AMH measurement. Data are divided into three gonadotoxicity groups, predicted mean log-transformed AMH trajectories over years since cancer treatment (bold lines with green for low, blue for moderate, red for high gonadotoxicity). Mean curves are truncated when the number of individual participants remaining in the group is fewer than 10. Individual log-transformed AMH levels (+) and predicted trajectories (short lines related to +) also are depicted in the same colour as their gonadotoxicity group. This figure is original and based on data from (35).

Variation in AMH levels by treatment also suggest that AMH may serve as a biomarker of gonadotoxicity. Acutely, between cancer diagnosis and end of treatment, AMH levels fell more in those exposed to alkylating chemotherapy exposure, compared to those not exposed to alkylators (32). Longitudinally, the rate of recovery in AMH did not vary by alkylator exposure, but this could be limited by sample size. Recently, using a hybrid cross-sectional and prospective cohort design, the post-treatment trajectory of AMH was modeled based on data from 763 patients with common cancers (**Figure 3**) (35). The magnitude of AMH recovery and duration of plateau was less for those whom underwent highly gonadotoxic therapies, compared to low or moderate gonadotoxic treatment groups. This study also used AMH measurements in dried blood spots collected at home: this technique may be of value to pursue large-scale clinically important questions.

### Post-Treatment AMH and Diagnosing Ovarian Insufficiency

The accurate diagnosis of permanent ovarian insufficiency is of considerable value in patients treated for cancer beyond fertility considerations. Ovarian function is also of importance in hormone dependent breast cancer as it may impact on choice of endocrine therapy. The use of aromatase inhibitors is now established to improve survival in post-menopausal women with breast cancer (52), but these drugs require concurrent ovarian suppression with GnRH agonists in pre-menopausal women. Uncertainty in identifying which women have become truly post-menopausal following treatment when they were pre-menopausal beforehand results in reluctance to stop GnRH agonist ovarian suppression even when in reality in an individual patient it may not be required. In the context of the normal menopause, initial studies showed that AMH became undetectable several years before the menopause, and therefore the assays available at that time were clearly insufficiently sensitive to have confidence in the use of AMH as an accurate diagnosis of POI following chemotherapy. However, currently available assays, both automated (Roche and Beckman Coulter) and the manual PICO assay (Ansh laboratories), have markedly improved sensitivity, and data on the relationship between AMH and natural menopause are becoming clearer (53), although age remains an important determinant of the accuracy of prediction. In a re-analysis of 98 blood samples taken 2 years after breast cancer treatment using the PICO assay, undetectable AMH was an extremely good predictor that ovarian function would not recover over the following few years, with 96% specificity (54). Subsequently the value of an undetectable AMH level, using the Roche automated assay, was shown to be an accurate diagnostic test for POI at 2 years following diagnosis with 100% sensitivity and 73% specificity (55), thus it appears that after allowing 2 years for any potential recovery of ovarian function, an undetectable AMH level is indeed an accurate index that recovery of ovarian function is very unlikely. While these data are exciting, it is important to note that substantial inter-assay differences remain with regard to AMH assays (56), which may be of particular importance at the lower limit of detection, and thus, the generalizability of cut points is assay-dependent. It is also the case that a proportion of women, perhaps as high as 10% in a young population (57), may have episodes of vaginal bleeding after more than 2 years of post-cancer amenorrhea, possibly reflecting transient ovarian activity.

AMH levels have been shown to be not influenced by tamoxifen co-administration, but they are suppressed by GnRH agonist administration over a period of several months (25), thus that needs to be taken into account in analyzing AMH levels in that context. Given the high predictive value of an undetectable AMH level at 2 years, the question is therefore how early after chemotherapy can AMH be used to accurately identify permanent POI. In that same analysis of women with breast cancer, AMH analysis at the end of chemotherapy was also analyzed (55). Overall, in a group of 68 women, an undetectable AMH at end of chemotherapy had a sensitivity of 78% and specificity of 82% for prediction of POI at 2 years, giving a diagnostic odds ratio of 10.9. This surpassed the value of FSH, which while it had a very high sensitivity (inevitable given that it is part of the diagnosis of POI), the specificity was low, and the diagnostic odds ratio was only 5.5. However, in a sub-group analysis of women aged over 40, the accuracy of AMH at end of treatment was improved with a sensitivity of 91% and specificity 82%, giving a high diagnostic odds ratio of 42.8. It may therefore be that using these high sensitivity assays that an AMH assessment on completion of chemotherapy can accurately identify those women who will not show any recovery of ovarian function following chemotherapy, and this may be of value in determining the most appropriate adjuvant endocrine treatment, but this may be limited to women in their forties. More conservatively, breast cancer survivors who are amenorrhoeic without GnRH agonist suppression for 2 years after chemotherapy and have an undetectable AMH level may be candidates for aromatase inhibitor endocrine therapy without concurrent GnRH agonist, but subsequent vaginal bleeding would require reassessment of ovarian function. This requires prospective evaluation.

## Conclusion

The above discussion clearly shows the value of pre- and post-treatment AMH in female cancer survivors for predicting post-treatment ovarian function, serving as a biomarker of treatment gonadotoxicity, and in the diagnosis of ovarian insufficiency, particularly when a sufficient time for early recovery of ovarian function has been allowed to elapse. Pretreatment analysis of AMH level is of value in predicting long term ovarian function in women with breast cancer but its value in other diagnoses and with other types of chemotherapy regimen, particularly when less gonadotoxic are unclear. The interaction with age in this respect is intriguing, and it is likely to require a greater understanding of these adverse effects of chemotherapy on the different compartments and cell types of the ovary. There are promising data that in certain sub-groups AMH may be of value shortly after completion of chemotherapy and ultimately this may be of value in guiding adjuvant endocrine therapy in some women with breast cancer. Current data suggest that AMH is not of value in predicting short term fertility in women following cancer treatment as shown both by specific analysis (58) and in individual cases within larger analyses (39), as is the case for women in the general population (59, 60). Unfortunately there remains a dearth of data regarding the use of AMH to estimate time to menopause in the post-cancer treatment setting and beyond that into whether AMH can help stage the process of reproductive senescence similar to in the general population (61). Data are lacking on AMH’s relationship to non-reproductive late effects relating to estrogen deficiency such as bone health and potentially cardiovascular and cerebral vascular function. Larger prospective studies with these diverse end points are needed to clarify the key areas where AMH is of value in this context.

## Author Contributions

All authors contributed to the article and approved the submitted version.

## Funding

The authors work in this field has been supported by MRC grant MR/N022556/1 (to RA) and NIH HD080952 (to HS).

## Conflict of Interest

RA has undertaken consultancy work for Roche Diagnostics.

The remaining author declares that the research was conducted in the absence of any commercial or financial relationships that could be construed as a potential conflict of interest.
